# Can Digital Economy Development Contribute to the Low-Carbon Transition? Evidence from the City Level in China

**DOI:** 10.3390/ijerph20032733

**Published:** 2023-02-03

**Authors:** Bei Liu, Yukun Li, Xiaoya Tian, Lipeng Sun, Pishi Xiu

**Affiliations:** 1 School of Management, Nanjing University of Posts and Telecommunications, Nanjing 210003, China; 2 Business School, Shandong Normal University, Jinan 250358, China; 3 School of Management, Wenzhou Business College, Wenzhou 325035, China

**Keywords:** digital economy, carbon emission intensity, “broadband China” strategy, innovation quality, policy linkage

## Abstract

As a new engine to promote high-quality development and a sustainable economy, the digital economy (DE) plays a key role in achieving carbon reduction targets. In this paper, we use the “broadband China (BC)” policy as a proxy variable for the DE and employ the panel data of Chinese prefecture-level cities from 2006 to 2019 to investigate the effect of DE development on carbon emission intensity and its mechanism of action. It is found that (1) DE development significantly reduces the carbon emissions of cities and presents dynamic and sustainable characteristics; (2) the results of mechanism tests indicate that DE development is more inclined to reduce carbon emission intensity by improving regional innovation quality than by improving regional innovation quantity; (3) the impact of DE development on carbon emission intensity differs among cities with different characteristic attributes and different environmental regulation intensity, and the emission reduction effect is more obvious in non-resource-based cities, cities with lower environmental regulation intensity, and cities with weaker environmental target constraints; (4) the impact of DE development and innovation-driven development strategies on reducing carbon emission intensity has a policy linkage effect.

## 1. Introduction

Relying on the comparative advantage of production factors, China has made significant achievements in promoting industrial structure transformation, deepening international cooperation, expanding trade scale, and enhancing the global competitiveness of China’s manufacturing industry. However, the long-standing crude development model has not only led to the low efficiency and weak technology of China’s economy, but it has also made the transformation of China’s economy into a green development model face serious challenges. At present, China’s economy is gradually moving towards high-quality development, and improving carbon productivity and reducing carbon emissions are important steps toward insuring the success of the “double carbon” goal. The digital economy (DE), with its strong resource allocation efficiency and high technological innovation, has broken through the traditional economic development model and become a viable path for high-quality development. Moreover, “carbon peak” and “carbon neutral” targets have brought new opportunities for China’s economy to shift to a high-quality and environmental-friendly development model, while also posing new challenges to the development efficiency and speed of China’s economy. The traditional economy is gradually transforming into a DE, which will help China’s cities’ low-carbon urban transformation [1].

In September 2020, China announced its carbon dioxide emissions peak target by 2030 and carbon neutral vision by 2060, putting forward a clear timetable for China to reduce carbon emissions and highlighting China’s ambition and role as a great power. In November 2021, the Ministry of Industry and Information Technology pointed out that the DE should be firmly promoted. Digital technology can alleviate the problem of the low efficiency of enterprise resource allocation [2,3,4,5] and enhance the resilience of China’s economic development, which can help build a new pattern of dual domestic and international cycles to promote each other [6,7]. Thus, with a booming DE, can China’s “BC” pilot policy help cities transform into low-carbon cities? Is there a significant causal relationship between the DE and pollution control and emission reduction? That is, can the implementation of the “BC” strategy significantly improve the status of carbon emissions? How can the intrinsic transmission mechanism of both be identified? Therefore, it is important to clarify the relationship between the DE and the low-carbon transformation in China in order to promote the DE and win the battle of environmental governance. Most existing studies focus on the impact of DE development on urban carbon emission reduction but do not pay attention to its internal transmission mechanism, nor do they analyze whether there is a differential impact on the urban low-carbon transition. On this basis, using the panel data of prefecture-level cities in China from 2006 to 2019, the relationship between the DE and carbon emission intensity and its mechanism are discussed, as are the heterogeneity effect of the DE on urban carbon emissions with different characteristics and intensities of environmental regulation. The innovation lies in the following aspects: first, it starts from BC policy and studies the urban low-carbon transition path of DE development from a new perspective; second, the effect of the DE on the carbon emission environment and its internal mechanism is discussed; third, not only is the carbon emission effect of DE policy discussed, but the synergistic effect of different policies on carbon emission is also emphasized.

The remaining structure is arranged as follows: Part II presents the literature review and research hypothesis; Part II introduces the econometric model setting and data sources; Part IV presents the empirical test to explore the DE and low-carbon development, combining heterogeneity and the mechanism perspective, and analyzing the linkage effect of BC pilot policy and innovative city pilot policy; Part V presents the discussion. Part VI is the conclusion and suggestions.

## 2. Theoretical Analysis and Research Hypothesis

### 2.1. Policy Background

Broadband network infrastructure construction is a national strategic public infrastructure to promote the high-quality development of the national economy. However, informatization construction is not accomplished overnight. In the early stage of broadband Internet construction, there are still many problems restricting the construction of broadband Internet. In August 2013, with the promulgation of the “BC” strategy and its implementation plan, the “BC” strategy was elevated from sectoral local development to the national level. At the national level, it also approved 120 cities as “BC” demonstration cities in three batches from 2014 to 2016 and pointed out that, with the support of specific policies, pilot areas should achieve significant improvement in regional broadband levels after the creation period of about three years. Indicators such as broadband access capacity, Internet penetration rate, and broadband user penetration rate have reached the leading level in China.

### 2.2. Theoretical Mechanisms and Research Hypotheses

The DE offers the possibility of efficient and high-quality development. Carbon reduction is the priority of the “double carbon” goal. This paper explores the relationship between the two, i.e., how the DE has contributed to China’s low-carbon transition and how it has manifested itself in terms of heterogeneity.

On 22 September 2020, China proposed that China will achieve “carbon peaking” by 2030 and “carbon neutrality” by 2060. The control of CO_2_ emissions and improving CO_2_ emission efficiency have become hot issues for scholars. The popularization of the Internet has brought the world into the digital age and created a series of digital development models such as the DE and the smart city. The Internet has also broken time-space restrictions, information barriers, and industrial boundaries in the traditional allocation of resources, forming a new digital impetus. Digital technology with environmental governance and green economic development has enabled the digital transformation of energy from production, supply, use and final pollutant emission, effectively alleviating the contradiction between the supply and demand of factors arising from regional environmental governance, and improving carbon emission performance while achieving a balance between the supply and demand of energy and resource consumption. As a critical initiative to promote digital technology, the “BC” strategy provides the necessary support to promote the low-carbon economy. The “BC” strategic pilot policy, as a critical initiative to promote digital technology, provides the necessary support to promote low-carbon economic development [8,9,10,11,12,13].

In recent years, some literature has used quasi-natural experiments to assess various effects of the DE. In particular, for “BC” demonstration cities, which are the focus of this paper, there are rich discussions in the existing literature, including the economic effects, technological effects, and industrial structure effects of “BC” demonstration cities [14,15,16,17]. For example, Zhang et al. (2022) examined the exogenous impact of broadband Internet infrastructure on business productiveness by using the implementation of “BC” strategy as an exogenous shock [18], and Zou and Pan (2022) used “BC” as a quasi-natural experiment to elucidate the effect of network construction on environmental pollution reduction using the double-difference method [9]. Li et al. (2022) studied how the development of the DE can reduce pollution emission, and this “carbon reduction effect” can be heterogeneous depending on the characteristics of the city’s location and the intensity of the environmental regulation [19]. Other literature has studied the low-carbon development path based on the DE context. Kalmaz and Kirikkaleli (2019) used digital technology to build a “smart city” and fully exploit renewable energy as a feasible way to achieve carbon emission reduction [20]. Huo et al. (2022) analyzed the impact of spatial externalities of DE development on energy efficiency and found that green finance can curb energy consumption [21]. Digital technology can help China’s carbon reduction process, and there is a long-term co-integration relationship between digitization, carbon emissions, innovation, and other key macroeconomic variables [22,23]. Digitalization has also dampened emissions figures everywhere, but the need for energy use as a result of economic growth has driven higher CO_2_ emissions in China. At the geographical level, the carbon emission efficiency of neighboring cities has a spatial spillover effect [24]. The literature on the evaluation of the policy effects of “BC” demonstration cities provides important ideas and insights. However, there is no literature that focuses directly on the impact of “BC” demonstration cities on carbon emissions, which provides room for this study.

The “BC” has a more significant effect on carbon emission performance improvement in non-resource-based cities [25,26]. Resource-based cities have a higher dependence on energy due to their industrial base. Although the widespread application of digital technology can alleviate regional environmental pressure, there is inertia in economic development, which to some extent makes it difficult to change the “brown” development model; therefore, the impact of the DE on carbon emission performance cannot be highlighted in resource-based cities [16,17]. The resource-dependent economic development model also has a differential impact on regional carbon emissions, with city size showing a positive correlation with regional economic growth to a certain extent, while China’s economic activities have distinct regional characteristics, with significant differences in location advantages, resource endowments, ecological environment, and ethnic and cultural dimensions in different regions. The DE reduces resource mismatch and improves resource efficiency, thus reducing the carbon emission intensity of enterprises [27,28]. The study found that pilot e-commerce policies reduced energy consumption. To some extent, e-commerce pilot policies have a greater effect on carbon emission reduction in non-resource-based cities [29].

The intensity of environmental regulation can lead to a differentiation in regional carbon emissions. Wang et al. (2022) examined the effectiveness of regional environmental regulation. Taking environmental regulation as an intermediary variable, they studied the impact of the DE on green technology innovation under the influence of environmental regulation [30]. The development of the DE promotes the improvement in the level of green technology innovation, and the DE can indirectly influence green technology innovation through the mediating variable of environmental regulation. The transmission of environmental regulation through digitalization and industrial structure innovation for green economy performance [31]. Local governments can strengthen digital application and technological innovation through environmental regulations, which is also a way to improve the efficiency of carbon emissions.

The DE could bring welfare effects in that it can drive the quality of innovation. Based on the new growth theory, technology innovation is a core element of knowledge. On the one hand, with the vigorous development of the DE in the new era, the transformation and upgrading of the traditional knowledge dissemination mode enables knowledge to be disseminated on a broader range and at a faster speed, The rapid spread of knowledge increases the knowledge stock at the social level, promotes the aggregation effect of innovative knowledge elements such as top talent, top technology enterprises, and scientific research capital, and improves the level of urban technological innovation [32,33,34]. On the other hand, the digital economy can effectively alleviate the information asymmetry problem among markets, form a more transparent and open market environment, and improve the efficiency of market operations [35].

**Hypothesis** **1.** *DE development has a significant carbon reduction effect*.

**Hypothesis** **2.** *The inhibitory effect of the DE on carbon emissions depends on the characteristics of urban attributes and the environmental regulations*.

**Hypothesis** **3.** *The carbon reduction effect of the DE is mainly realized by promoting the quality improvement of innovation*.

## 3. Study Design

### 3.1. Measurement Model

The double-difference method (commonly known as the multiplicative difference method) can primarily alleviate the endogeneity problem under the premise of satisfying its identification assumptions. Compared with other causal inference methods, differential model design is simpler and easier to operate. Therefore, the method has gradually become one of the most widely used current measures.

Based on the existence of the above advantages of double difference, it is important to further explore the natural impact effect of DE construction on regional carbon emission intensity. Using BC strategy as a quasi-natural experiment, the BC pilot cities are used as the treatment group and the non-pilot cities as the control group [36]. The specific model was constructed as follows:

(1) $$uco2_{it}=\alpha +\beta numecon_{it}+\gamma x_{it}+\eta_{i}+\nu_{t}+\varepsilon_{it}$$

$uco2_{it}$ is the explanatory variable, which indicates carbon intensity; $numecon_{it}$ is the core explanatory variable, which is assigned a value of 1 if a city has implemented BC policy in a certain year, and 0 otherwise; $x_{it}$ is the set of control variables, including economic development level, education level, financial development level, import and export trade, and foreign investment introduction; $\eta_{i}$ indicates individual fixed effects; $\nu_{t}$ indicates time-fixed effects; $\varepsilon_{it}$ indicates the unobservable random disturbance term.

### 3.2. Data Sources and Variable Selection

#### 3.2.1. Data Source

This paper selected data from 283 prefecture-level cities from 2006 to 2019 to study the impact of BC policy on regional carbon emission intensity. Unless otherwise specified, the data are taken from the China Urban Statistical Yearbook. Some missing data are made up of each city’s statistical yearbook.

#### 3.2.2. Variable Selection

**Figure 1:**
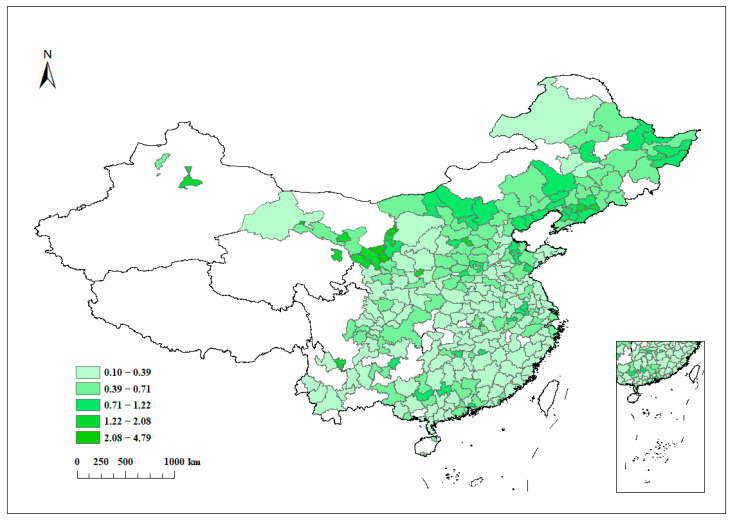
Average carbon emission intensity in 2006–2019.

(1) Explanatory variable: carbon emission intensity (*uco2*), which is constructed by referring to Canadell et al. (2007) [37] and Roberts and Grimes (1997) [38], using carbon emissions per unit of GDP as a proxy for carbon emission intensity. Figure 1 is the average carbon emission intensity in 2006–2019. It can be found that the carbon emission intensity is higher in northeast and central China.
(2) Core explanatory variable: BC strategy (*numecon*), based on the list of “BC” demonstration cities (city clusters) as the basis for dividing the treatment and control groups; cities that have become BC demonstration cities in the current year are assigned a value of 1, otherwise they are assigned a value of 0.
(3) Control variables: Referring to previous related studies [39,40], the following control variables are selected: the level of economic development (*lngdp*) is characterized by the logarithm of total GDP; the level of education (*edu*) is expressed by the logarithm of education expenditure in fiscal expenditure; government concern (*gov*) is expressed by the logarithm of the amount of government fiscal expenditure; the level of financial development (*fina*) is expressed by taking the logarithm of the year-end amount of deposit and loan balance; import and export trade (*trade*) is characterized by taking the logarithm of the total import and export trade; foreign investment introduction (*fdi*) is expressed by taking the logarithm of the amount of foreign direct investment. Table 1 shows the descriptive statistics of the above variables.

## 4. Analysis of the Empirical Results

### 4.1. Baseline Regression Analysis

To confirm the accuracy of the theoretical analysis, this section further quantitatively assesses the effect of BC policy implementation on regional CO_2_ emissions through a double-difference model with the following benchmark results.

Columns (1) and (2) of Table 2 report the effects of the implementation of BC strategy on carbon emission intensity. From the significance levels and signs of the empirical results, it is clear that BC strategy reduces the regional carbon emission intensity at the 1% significance level. Accordingly, the previous theoretical analysis is proved to be reliable.

### 4.2. Identification of the Condition Test

The above benchmark regression proves that BC strategy effectively reduces regional carbon emission intensity; however, we need to perform robustness tests to ensure the robustness of the results. This paper argues that the robustness test should be distinguished into two parts, i.e., before directly conducting the robustness test on the robustness of the empirical results, the identification effect test on the model validity should be conducted. Only if the model validity is satisfied, then the coefficients estimated from the model may be robust. Based on this, this paper first conducts an identification effect test on the model, which is divided into two parts as follows: a parallel trend test and a sample selection bias test.

#### 4.2.1. Parallel Trend Test

The parallel trend is the essential prerequisite assumption for the use of double difference. Maintaining the strict exogeneity of the policy requires that there should be no significant difference between the experimental and control groups themselves before the real policy takes place. Only then will the estimates we obtain using the double-difference method be consistent estimates. On the basis of this, the specific model of this paper is constructed as follows:

(2) $$uco2_{it}=\alpha +\overset{5}{\underset{k=-6}{\sum }}\beta_{k}numecon_{it}{}^{k}+\gamma x_{it}+\eta_{i}+\nu_{t}+\varepsilon_{it}$$

where $numecon_{it}{}^{k}$ is a series of dummy variables, and $numecon_{it}{}^{k}$ is assigned with the following rules: $n_{i}$ is the year in which city *i* implemented BC policy ($n_{i}$ = 2014, 2015, 2016). If *t* − $n_{i}$ ≤ −6, then define $numecon_{it}{}^{-6}$ = 1; if *t* − $n_{i}$ = k, then define $numecon_{it}{}^{k}$ = 1; if *t* − $n_{i}$ ≥ 5, then define $numecon_{it}{}^{5}$ = 1; other variables are set as the benchmark.

**Figure 2:**
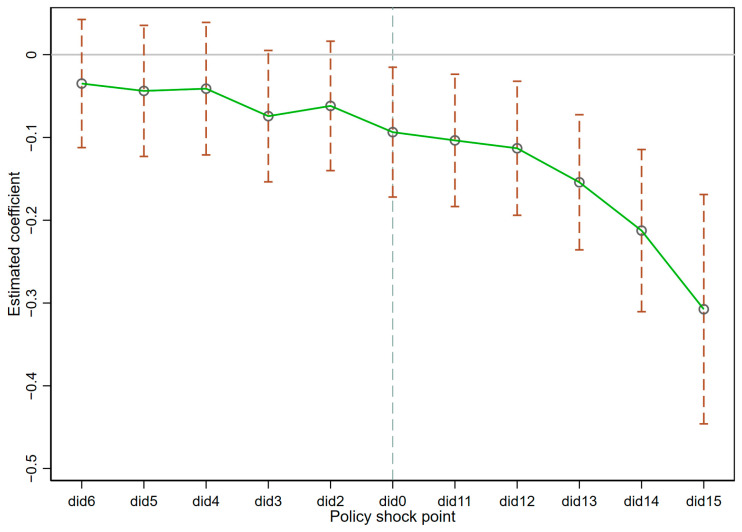
Parallel trend test.

Figure 2 shows the parallel trend results. The estimated coefficients of the pseudo-policy shocks before the occurrence of the policy $\beta_{k}$ are not significant, which indicates that the pseudo-policy shocks did not affect the regional carbon emission before the occurrence of the real policy, which satisfies the parallel trend hypothesis. In addition, the estimated coefficient $\beta_{k}$ is significantly negative after the occurrence of the actual policy, and the negative value becomes more prominent each year, which further indicates that: BC strategy significantly reduces the carbon emission of the pilot region after the implementation of the real policy.

#### 4.2.2. Sample Selection Bias Test

The country may consider exogenous factors such as the region’s economic base, network infrastructure status, and location advantages in the selection of pilot areas, which in turn leads to the existence of a sample selection bias problem and eventually affects the robustness of this paper. Therefore, when testing the validity of the double-difference model, it is essential to consider not only the parallel trend test, but also to further exclude the problem of sample selection bias or the problem of non-random grouping in the policy. Based on this, this paper adopts a PSM-DID model with 1:1 caliper nearest-neighbor matching to eliminate the sample selection bias and grouping non-randomness problems.

**Figure 3:**
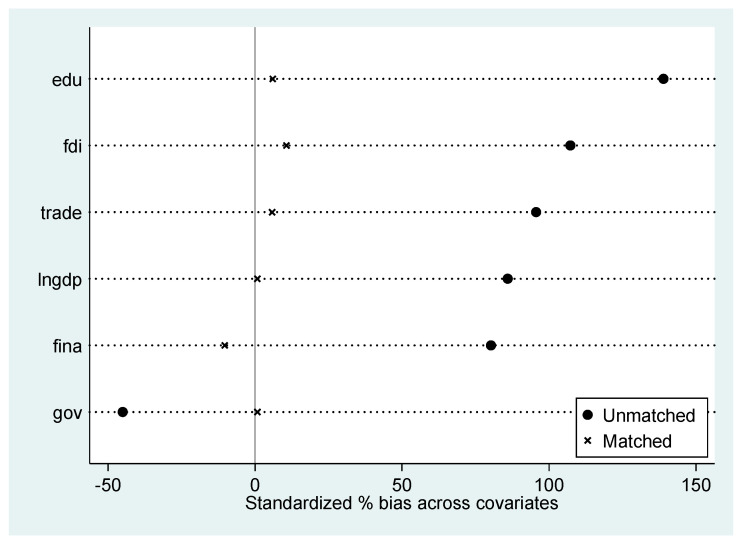
Propensity score matching effect.

Columns (1) and (2) of Table 3 represent the effects of the implementing of BC strategy on regional carbon emissions without and with the inclusion of control variables after propensity score matching. With propensity score matching to obtain a new control group, the implementing of BC strategy still reduces the regional carbon emission intensity at the 1% significance level regardless of the inclusion of control variables. The result indicates that the conclusions of the benchmark regression are robust, even if the possible sample selection bias of the model is eliminated. In addition, Figure 3 of the propensity score matching effect shows that the samples of covariates that are not matched are significantly different from the zero value, while the samples of covariates that are matched are all perturbed around the zero value, further verifying that the benchmark results are robust.

### 4.3. Robustness Test of the Empirical Results

#### 4.3.1. Placebo Test

To further verify that the significant reduction in carbon emission intensity after the implementing of BC strategy is brought about by the real policy and not by other time-varying factors, this paper draws on Topalova’s (2010) treatment: a placebo test with transformed policy time points is used to validate the baseline empirical results [41]. This is done by randomly constructing 1000 sets of pseudo-policy shock time points and conducting regressions identical to the benchmark, then calculating the estimated coefficients and *p*-values of the core explanatory variables corresponding to the 1000 pseudo-regressions. If the estimated coefficients of the core explanatory variables of the pseudo-regressions are not significant, it means that the carbon emission intensity of BC pilot cities is indeed brought about by the real policy rather than other time-varying factors. If the results are opposite to the above expected results, it proves that carbon emission intensity is not necessarily caused by BC, and there may be interference from other factors.

**Figure 4:**
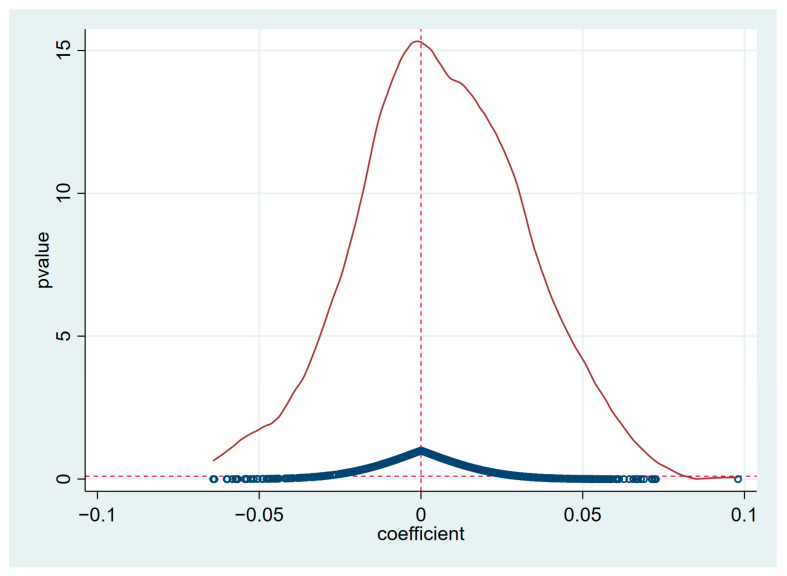
Placebo test.

The solid red line in Figure 4 indicates the kernel density curve of the estimated coefficients of the core explanatory coefficients of the 1000 pseudo-regressions; the blue hollow circles are the *p*-values of the 1000 pseudo-regressions; and the red dashed line at the level of the horizontal axis indicates *p* = 0.1. Based on this, it is obvious from Figure 4 that, first, the estimated coefficients of the core explanatory variables of the 1000 pseudo-regressions are all clustered around the value of 0, and second, the majority of *p*-values are distributed above the dashed line at *p* = 0.1. The above results indicate that the carbon emission of BC is indeed brought about by real policies rather than by other time-varying factors.

#### 4.3.2. Control Other Policy Interference

**Figure 5:**
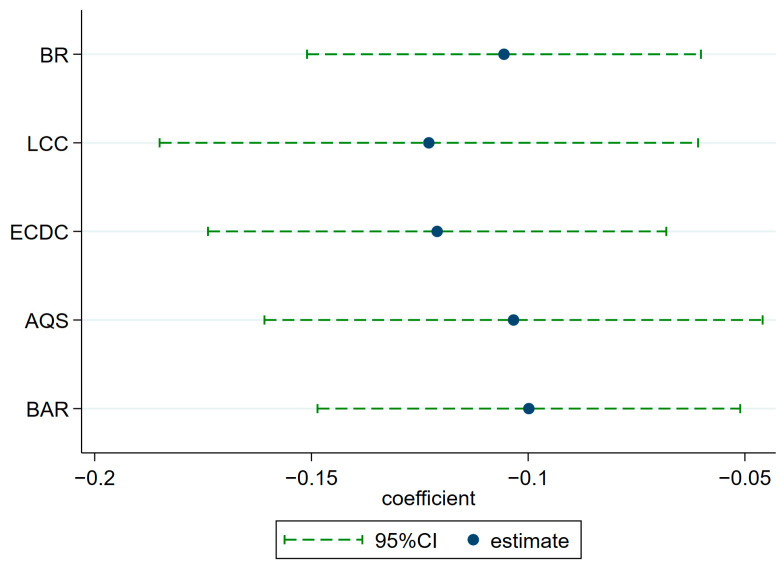
Controlling for other policy disruptions.

As China’s domestic environmental concerns continue to grow, the country has enacted a series of environmental regulatory policies and has imposed higher environmental requirements on important national strategic policies in the areas of trade and international cooperation already in place. These environmental regulatory and trade and international cooperation policies include Low-Carbon Pilot Cities (LCC), Ecological Civilization Demonstration Cities (ECDC), Ambient Air Quality Standards(AQS), and the Belt and Road Initiative (BAR), respectively. It has also been established that these policies can have a significant impact on regional carbon emissions. From the above statements, it is clear that all of the above policies may have an impact on regional CO_2_ emissions, which in turn affects the benchmark results. Therefore, policy dummy variables for the above policies are constructed separately in this paper and put into the model as control variables in turn for regression. Obviously, the empirical results displayed in Figure 5 indicate that the implementation of BC strategy still significantly decreases regional carbon emissions while controlling for the abovementioned types of policy disturbances in turn, which further proves that the benchmark results are robust.

#### 4.3.3. Other Robustness Tests

Although the double-difference method can alleviate most of the endogeneity problems under the condition that the identification assumption is satisfied, to further improve the robustness of the results and eliminate possible endogeneity problems, this paper adopts the slope index, which represents the characteristics of urban landforms, as the instrumental variable of BC policy for further empirical research. The urban slope index is chosen as an instrumental variable for BC policy because it satisfies two conditions of an instrumental variable, namely, the correlation hypothesis and the exogeneity hypothesis. From the relevance hypothesis, the magnitude of urban slope affects the cost of network infrastructure construction and also affects the transmission of broadband network signals and thus the implementation effect of BC strategy; from the exogeneity hypothesis, urban slope, as a natural geographical variable, basically does not affect the regional carbon emission intensity. Here, we use urban slope cross-sectional data interacted with temporal dummy variables as the actual instrumental variables added to the model.

The empirical results of the regression using two-stage least squares are reported in Column (1) of Table 4, and the findings show that the F value of 22.89 in the first stage is much greater than 10, and the F statistic is greater than the critical value at the 10% bias level audited by Stock and Yogo, indicating that the instrumental variables are highly correlated with the core explanatory variables and can be ruled out. BC strategy can still effectively decrease regional carbon emission intensity under the condition of further eliminating the endogenous influence of the model.

According to the above variable descriptions, we can see that the carbon emission index is characterized by the carbon emission per unit of GDP in this paper, and the index construction is relatively singular, to avoid the problem of biased results due to the index measurement method. To avoid the bias of the results caused by the index measurement, this paper takes the logarithm of the total carbon emission of cities as a proxy variable of carbon emission. Further, it investigates the effect of the implementation of BC strategy on regional carbon emission. Study results are shown in Column (2) of Table 4: with lnco2 as the explanatory variable, the estimated coefficient is still significantly negative at the 1% level. The previous identification condition test has demonstrated that the model passes the parallel trend test. However, rigorously, the previous dynamic effects test only demonstrates that the model passes the ex-ante trend test due to the unobservable counterfactual values in the ex-post treatment group. Therefore, in order to ensure as much as possible that the ex-ante parallel trend is also satisfied after the policy occurs, this paper includes the interaction terms of the control variables with the time variables and the interaction terms of the control variables with the time polynomials, respectively, in the model. The purposes are as follows: to eliminate the changes in the control variables at the time level so that the control variables are consistent before and after the occurrence of the policy. The parallel trends that exist ex-ante are guaranteed to continue further after the occurrence of the policy, thus obtaining a clean policy treatment effect. Columns (3) and (4) of Table 4 report the results for adding the control variable to the model with the time variable interaction term and adding the control variable to the model with the time polynomial interaction term, respectively. BC strategy is still effective in reducing the regional carbon emission intensity, controlling for the time trend. In addition, in order to further exclude other unobservable time-varying factors caused by long sample intervals and the existence of sample extremes in the data itself that affect the dependability of the empirical results, the paper shortens the sample intervals and shrinks the tails of the data, respectively, in columns (5) and (6) of Table 4. Obviously, after shortening the sample interval and tailing the data, the implementing of BC policy still significantly decreases the regional carbon emission, which further indicates that the baseline results are reliable.

### 4.4. Heterogeneity Analysis

#### 4.4.1. Heterogeneity of City Characteristics and Attributes

The resource-dependent economic development model also has a differential impact on regional carbon emissions. The size of cities shows a positive correlation with regional economic growth to a certain extent, while China’s economic activities have distinct regional characteristics, and different regions have obvious differences in terms of location advantages, resource endowment, ecological environment, and ethnic and cultural dimensions. Therefore, this section tests the differential impact of the DE and carbon emissions based on different characteristics of cities as attributes; Columns (1) and (2) of Table 5 shows the differential impact of “BC” policy on regional carbon emission levels in resource-based cities and non-resource-based cities. The influence of “BC” policy on regional carbon emissions in resource-based cities is significantly negative at 5%, while the emission reduction effect of “BC” policy on non-resource-based cities is significantly positive at 1%, indicating that the impact of “BC“ policy on regional carbon emissions is significantly negative at the 5% level. The reason is that resource-based cities are bound by resources and overly dependent on resources, and it is difficult to change their traditional resource-dependent development mode by relying on the development of the DE alone, and it is difficult to achieve a significant carbon emission reduction effect. The results of the city size sub-sample regression are shown in columns (3) and (4) of Table 5. Under the sample of large and medium-sized cities, BC strategy reduces the regional carbon emissions. In contrast, BC strategy does not have a significant impact on regional carbon emissions under the small city sample. From Columns (5), (6) and (7), we can see that, relative to western cities, BC strategy in eastern and central cities effectively reduces the regional carbon.

#### 4.4.2. Heterogeneity in the Intensity of Environmental Regulation

The visible hand of the government plays a self-evident role in China’s economic development. Therefore, in addition to considering that differences in location and city size may lead to heterogeneity in policy effects, it is also necessary to further consider whether government environmental regulation, which is considered as the tangible hand of Keynes, may also make policy effects heterogeneous in impact. This paper uses the frequency of environmental protection terms appearing in the annual government work reports of each prefecture-level city to measure local government environmental goal constraints. Integrated utilization rate of industrial solid waste is used to measure the degree of environmental regulation, and when the environmental regulation is stricter, the waste utilization rate is high, and vice versa when the utilization rate is low. The regression results of environmental target constraint heterogeneity are shown in columns (1) and (2) of Table 6; the impact of DE development on regional carbon emissions is significantly negative at the level of 5% in regions with higher constraints on environmental goals, while the effect of DE development on regional carbon emissions is significantly negative at the 1% level in areas with low environmental target constraint, which means that the carbon reduction effect of the DE in areas with low environmental target constraint is more significant. The regression results of environmental regulation heterogeneity are shown in columns (3) and (4) of Table 6; in areas with high environmental regulation, the regression coefficient of the DE on carbon emissions is significantly negative at the level of 10%, while the regression coefficient of the DE on carbon emissions is significantly negative at the 1% level in low environmental regulation areas. Due to high environmental target constraint and environmental regulation areas, in the face of higher government pressure, localities have achieved effective control over carbon emissions, and the inhibitory effect of the DE on carbon emissions is no longer prominent. The reduction of carbon emissions in low environmental goal constraint and low environmental regulation areas is not because of the government, but mainly due to the change of the city’s own economic development mode and the development of science and technology so it can effectively achieve a reduction in carbon emissions.

### 4.5. Intermediary Effect Test

Based on the previous section, we have learned that BC policy mainly reduces regional carbon emissions by improving the quality of regional innovation and promoting advanced industrial structures. To confirm the reliability of the theoretical analysis, this paper refers to the study of Akerman et al. [42] and further adds the interaction terms of mediating variables and core explanatory variables in the baseline model (1) to construct the following four models to carry out the mechanism test.

u*co*2_*it*_ = *α* + *ρ*_1_*numecon_it_* × *patentnum_it_* + *ρ*_0_*numecon_it_* + *φpatentnum_it_* + *γx
_it_* + *η_i_* + *ν_t_* + *ε_it_* (3)

u*co*2_*it*_ = *α* + *β*_1_*numecon_it_* × *patentqunl_it_* + *β*_0_*numecon_it_* + *λpatentqunl_it_* + *γx
_it_* + *η_i_* + *ν_t_* + *ε_it_* (4)

In particular, it should be noted that the patentnum variable in model (3) indicates the number of regional innovations, which is expressed using the total number of regional patent applications; the patentqunl variable in model (4) indicates the regional patent quality, which is characterized by the number of regional patent applications for inventions as a percentage of the total number of patent applications [43].

Table 7 reports the empirical results, where columns (1) and (2) and columns (3) and (4) report the effects of innovation quantity and innovation quality as mediating effects of BC strategy to decrease carbon emissions with and without the inclusion of control variables, respectively; we focus on the magnitude and significance level of the coefficients of the interaction term between the policy dummy variables and the mediating variables. From Table 7, we obviously know that, firstly, from the innovation channel, the coefficients of the interaction terms of innovation quantity and innovation quality with the policy dummy variables are significantly negative at 5% and 1% significance levels, respectively, which indicates that BC strategy can reduce urban carbon emission by enhancing regional innovation quantity and innovation quality at the same time, but from the interaction term significance levels and coefficient magnitudes, we can further find that the DE is more inclined to reduce carbon emissions by improving the quality of innovation than by improving the quantity of innovation.

### 4.6. Policy Linkage Effect

BC policy is an important way for China to achieve carbon neutrality, and the innovation city pilot policy, as one of the national policies to promote the level of regional innovation, will also reduce carbon emissions. Therefore, it is important to bring into play the linkage between BC pilot policy and the innovation city pilot policy to break the limitations of the region’s own development and to accelerate the promotion of regional emission reduction.

Based on this, this section investigates whether the construction of BC drives the carbon-reduction effect of pilot regions on the regions involved in innovative cities. Specifically, a triple difference model is constructed as follows:

(5) $$uco2_{it}=\alpha +\beta didld_{it}+\gamma x_{it}+\eta_{i}+\nu_{t}+\varepsilon_{it}$$

It should be noted that the *didld* in model (5) is a triple interaction of the BC interaction term with the innovation city pilot region dummy variable. The model focuses on the  β coefficient, which, if significantly negative, indicates that BC construction has a policy linkage effect with the innovation city pilot policy in reducing regional carbon emission intensity.

Columns (1) and (2) of Table 8 are the regression results of the linkage effect of BC and innovative city pilot policy, and Column (2) adds the relevant control variables to Column (1), and the regression results show that the estimated coefficient of the triple interaction term *didld* is significantly negative at the 1% level, showing that BC and innovative city pilot policy significantly reduced the carbon emission of the areas covered by BC construction. In summary, it can be seen that effectively playing the linkage between BC and innovative city construction and promoting BC construction can help realize the local carbon emission reduction effect.

## 5. Discussion

The empirical results of the DE and urban carbon intensity emissions are discussed above. The empirical results are explained as follows: the DE promotes the double improvement of regional innovation quality and quantity through resource agglomeration, so as to reduce urban carbon intensity. Based on the heterogeneity analysis of urban attributes, resource-dependent cities tend to have resource advantages due to the difference in urban resource endowment, and the development of the DE will make resource-based cities weaker than non-resource-based cities in reducing carbon emission intensity. The size of a city will bring a stronger resource agglomeration effect. Therefore, the development of the DE in big cities will significantly reduce carbon emission intensity, while that in small cities will not. At the regional level, the central and eastern cities have a higher level of economic development, and the development of the DE started early, so the DE has a significant effect on urban carbon emission reduction, while the western cities have no significant impact on urban carbon emission reduction due to regional development factors. In the heterogeneity analysis based on environmental regulation intensity, due to the role of government in economic development, if there are higher environmental target constraints or higher environmental regulation intensity in a region, the government will play a greater role in promoting regional carbon emission reduction, thus offsetting the impact of the DE on urban carbon emission reduction. Therefore, in areas with low environmental target constraints and low environmental regulation intensity, DE development will significantly reduce urban carbon emission intensity. In the mechanism analysis of this paper, the DE promotes the improvement of the innovation level through resource integration and agglomeration and reaches the carbon emission reduction target through the transformation of achievements in innovation. In the analysis of the linkage effect of policies, the linkage effect of carbon emission reduction between pilot broadband cities and innovative cities is analyzed. Pilot policies of innovative cities can promote the improvement of regional innovation level and further promote regional carbon emission reduction. Therefore, pilot policies of BC and innovative cities can significantly reduce the carbon emission intensity of the regions covered by the construction of BC.

## 6. Conclusions

Building a digital power and winning the battle against pollution are important strategic initiatives for China to promote solid economic development. However, the intrinsic linkage between these two is yet to be explored. In this context, this paper studies cause and effect and the underlying mechanism between promoting the development of the DE and improving environmental pollution in a more systematic way based on “BC” strategy and urban panel data from 2006 to 2019 using the double-difference method, heterogeneity test, and mediating effect test. The main findings are as follows: first, advancing the DE can improve carbon emissions. After excluding other policy interference and sample selection bias, the implementation of “BC” strategy still effectively reduces CO_2_ emissions. In addition, the emission reduction effect gradually increases, showing the characteristic of “thick and thin”. Second, the heterogeneity analysis shows that the difference in urban attributes and environmental regulations will make the “BC” policy asymmetric in promoting the DE and improving carbon emission reduction, and the emission reduction effect is significant in non-resource-based cities, larger cities, cities located near the central-eastern part of the country, and cities with lower environmental regulation intensity. Carbon emission reduction in non-resource cities, cities of smaller size, cities geographically close to the west, and cities with higher environmental regulations is more significant, but the emission reduction effect of promoting the DE for pollution control of resource-based cities, cities of smaller size, cities geographically close to the west, and cities with higher environmental regulations is not significant. Third, further discussion of the underlying mechanism reveals that “BC” strategy reduces regional carbon emission intensity mainly by improving the quality of regional innovation, rather than by increasing the quantity of innovation and promoting the rationalization of industrial structures. The limitations of this paper are that it only focuses on the impact of DE development on carbon emissions at the city level, without studying the impact of carbon emissions at the enterprise level. In addition, an all-round DE index system can be constructed to measure DE development. Although the differences-in-differences model can solve the endogeneity problem to a certain extent, there is no appropriate exogenous instrumental variable to better solve the endogeneity problem, which is also an area for further study in the future.

The article suggests the following: first, the implementation of “BC” strategic deployment helps achieve the dual carbon goal. The DE is not only a new engine for improving economic development, but it is also a new idea for promoting development of society and grasping the opportunities of digital development at the new historical starting point. The DE has a long way to go, and it needs to continuously strengthen the foundation of development, activate the vitality of data factor production, construct the pillars of development, enhance the effectiveness of governance, expand the space of digital development, promote the improvement of innovation quality upgrades with the DE, expand the social welfare scope of the DE, and build the double cycle of digital industry and industrialized digitalization. For the relatively economically backward regions, it is more important to fully grasp the opportunities of DE development, improve the green economic infrastructure, complete the transformation to new green kinetic energy, and realize the beautiful vision that the silver mountain of gold is also the green mountain of water.

Second, we should combine the DE with the real economy as the main task through the release of DE technical innovation, the convergence of the multiplier effect, implementing backbone enterprises, key industries, industrial parks, and industrial clusters, implementing the digital transformation of key industries and the digital transformation of the support service of ecological cultivation projects, and promoting improvements in the quality of industries. The process should also include industrial upgrading using a digital background for dynamic supervision through the market reform of data elements, improving market rules and institutions, stimulating digital vitality, and constantly promoting innovation in the industrial structure. In addition, the government needs to consider the local resource endowment and environmental regulations in order to push the comprehensive development of the DE and the reasonable reduction of environmental problems.

Third, improving the quality of regional innovation reduces carbon emissions. The DE promotes the low-carbon transformation of cities through innovation. It is necessary to introduce green finance into innovation technology by taking the breakthrough of core innovation technology as the key point, protecting the achievements of technology transformation, and further promoting low-carbon transformation in cities. Enrich the diversity of green financial products in the green innovation market, develop hierarchical categories of financial incentive grants and green credit services, ensure smooth circulation of funds to support green technology R&D, guarantee green innovation technologies from conception to implementation, and increase the speed of green innovation transformation. Create a green market environment with fair competition, sound systems and regulations with scientific and appropriate policy support; promote green innovation activities in the economy in all aspects and at multiple levels; and eliminate the innovation market problems of being too tight, too stuck, and too costly. Cultivate green innovation and low-carbon industrial talent teams, promote green technology infrastructure projects, and ultimately help achieve the dual carbon goal.

## Figures and Tables

**Table 1:** Descriptive statistics.

Variable	Implication	Obs	Mean	Std. Dev.	Min	Max
*uco2*	Carbon emission intensity	3962	0.544	0.61	0.023	9.612
*lngdp*	Economic development level	3962	10.416	0.724	4.595	13.056
*edu*	Education level	3962	3.549	1.313	0	7.051
*gov*	Government attention	3962	0.18	0.099	0.035	1.027
*fina*	Level of financial development	3962	0.885	0.56	0.075	9.622
*trade*	Import and export trade	3962	13.676	2.151	2.773	19.648
*fdi*	Introduction of foreign investment	3962	9.472	2.623	0	14.941
*patentqunl*	Innovation quality	3962	0.108	0.075	0	0.5
*patentnum*	Number of innovations	3962	6.682	1.798	1.386	12.02
*numecon*	Policy dummy variables	3962	0.128	0.334	0	1

**Table 2:** Baseline regression results.

	(1)	(2)
	*uco2*	*uco2*
*numecon*	−0.108 ***	−0.107 ***
	(−4.73)	(−4.64)
*lngdp*		−0.196 ***
		(−5.47)
*edu*		0.040
		(1.45)
*gov*		0.937 ***
		(5.59)
*fina*		0.048 **
		(2.43)
*fdi*		0.017 ***
		(3.05)
*trade*		0.042 ***
		(3.66)
*Year FE*	Yes	Yes
*City FE*	Yes	Yes
*Constant*	0.762 ***	1.688 ***
	(37.79)	(4.71)
*N*	3962	3962
*R^2^*	0.153	0.178

*Notes: t*-statistics in parentheses, *** *p* < 0.01, ** *p* < 0.05.

**Table 3:** PSM-DID.

	(1)	(2)
	*uco2*	*uco2*
*numecon*	−0.104 ***	−0.084 ***
	(−5.11)	(−4.15)
*lngdp*		−0.237 ***
		(−6.11)
*edu*		0.030
		(0.96)
*gov*		1.759 ***
		(8.69)
*fina*		−0.032
		(−1.63)
*fdi*		−0.009
		(−1.29)
*trade*		0.041 ***
		(3.54)
*Year FE*	Yes	Yes
*City FE*	Yes	Yes
*Constant*	0.749 ***	2.302 ***
	(35.49)	(6.08)
*N*	3255	3255
*R* ^2^	0.210	0.253

*Notes: t*-statistics in parentheses, *** *p* < 0.01.

**Table 4:** Robustness tests for each category.

	(1)	(2)	(3)	(4)	(5)	(6)
	Instrumental Variable	Replacing the Explanatory Variable	Control Time Trends		Shortened Sample Intervals and Data Tailoring	
	*uco2*	*uco2*	*uco2*	*uco2*	*uco2*	*uco2*
numecon	−0.089 ***	−0.125 ***	−0.074 ***	−0.078 ***	−0.062 ***	−0.072 ***
	(−3.21)	(−5.07)	(−3.13)	(−3.31)	(−2.65)	(−4.25)
*lngdp*	−0.193 ***	0.375 ***	−0.337 ***	−0.099 *	−0.419 ***	−0.160 ***
	(−2.98)	(9.72)	(−4.22)	(−1.83)	(−8.37)	(−5.12)
*edu*	0.041	0.125 ***	0.026	−0.004	0.026	0.068 ***
	(1.61)	(4.19)	(0.72)	(−0.13)	(0.69)	(3.25)
*gov*	0.958 ***	0.501 ***	0.783 **	−0.720	0.881 ***	1.040 ***
	(4.43)	(2.79)	(2.29)	(−1.47)	(3.77)	(7.43)
*fina*	0.047	−0.016	0.052 *	0.482 ***	0.056 ***	0.015
	(1.19)	(−0.76)	(1.72)	(7.70)	(2.78)	(0.69)
*fdi*	0.016 *	0.010 *	−0.001	0.038 ***	0.014 **	0.008 *
	(1.71)	(1.66)	(−0.13)	(3.34)	(2.47)	(1.91)
*trade*	0.041 **	0.084 ***	0.070 ***	−0.015	0.034 **	0.037 ***
	(2.29)	(6.88)	(3.73)	(−0.98)	(2.40)	(4.23)
*Year FE*	Yes	Yes	Yes	Yes	Yes	Yes
*City FE*	Yes	Yes	Yes	Yes	Yes	Yes
*Phase I F-value*	22.89					
*Kleibergen-Paap rk Wald F-statistic*	1777.67					
*Constant*	1.480 **	0.374	3.068 ***	1.325 ***	3.892 ***	1.379 ***
	(2.44)	(0.97)	(3.49)	(2.65)	(6.93)	(4.54)
*N*	3962	3962	3962	3962	2547	3962
*R* ^2^	0.722	0.622	0.250	0.226	0.227	0.258

*Notes: t*-statistics in parentheses, *** *p* < 0.01, ** *p* < 0.05, * *p* < 0.1.

**Table 5:** Heterogeneity of city characteristic attributes.

	(1)	(2)	(3)	(4)	(5)	(6)	(7)
	Heterogeneity of Resource Endowments		City Scale Heterogeneity		Regional Heterogeneity		
	Non-Resource-Based Cities	Resource-Based Cities	Large and Medium-Sized Cities	Small Cities	East	Central	West
	*uco2*	*uco2*	*uco2*	*uco2*	*uco2*	*uco2*	*uco2*
*kdzg*	−0.076 **	−0.113 ***	−0.071 ***	−0.049	−0.099 ***	−0.114 ***	−0.102
	(−2.01)	(−3.89)	(−2.90)	(−1.02)	(−3.98)	(−4.47)	(−1.54)
*lngdp*	−0.079	−0.356 ***	−0.166 ***	−0.855 ***	−0.090 **	0.075	−0.269 ***
	(−1.64)	(−6.65)	(−4.25)	(−10.77)	(−2.07)	(1.30)	(−3.16)
*edu*	0.109 ***	−0.030	0.006	−0.096 **	0.037	0.095 ***	−0.056
	(2.86)	(−0.77)	(0.19)	(−2.19)	(1.21)	(2.78)	(−0.74)
*gov*	0.581 ***	1.538 ***	0.623 ***	0.026	0.936 ***	2.790 ***	0.806 **
	(2.62)	(6.20)	(2.78)	(0.10)	(4.42)	(7.90)	(2.34)
*fina*	0.034	0.033	0.087 ***	0.023	0.174 ***	−0.044 **	0.046
	(1.43)	(0.99)	(3.00)	(0.86)	(5.95)	(−2.25)	(1.00)
*fdi*	0.026 ***	0.007	0.019 ***	0.016 *	−0.024 ***	0.018 *	0.035 ***
	(3.69)	(0.91)	(2.98)	(1.83)	(−3.30)	(1.81)	(3.23)
*trade*	0.014	0.065 ***	0.012	0.039 **	0.124 ***	−0.008	0.017
	(0.81)	(4.22)	(0.87)	(2.16)	(7.91)	(−0.50)	(0.67)
*Year FE*	Yes	Yes	Yes	Yes	Yes	Yes	Yes
*City FE*	Yes	Yes	Yes	Yes	Yes	Yes	Yes
*Constant*	0.890 *	3.088 ***	2.036 ***	7.923 ***	−0.256	−0.733	2.974 ***
*N*	(1.85)	(5.80)	(4.90)	(10.54)	(−0.55)	(−1.35)	(3.42)
*R^2^*	1456	2506	2702	1260	1680	1120	1162

*Notes: t*-statistics in parentheses, *** *p* < 0.01, ** *p* < 0.05, * *p* < 0.1.

**Table 6:** Heterogeneity of environmental regulation intensity.

	(1)	(2)	(3)	(4)
	High Environmental Target Constraints	Low Environmental Target Constraints	High Environmental Regulation Intensity	Low Environmental Regulation Intensity
	*uco2*	*uco2*	*uco2*	*uco2*
*kdzg*	−0.069 **	−0.122 ***	−0.032 *	−0.275 ***
	(−1.97)	(−3.60)	(−1.84)	(−4.24)
*lngdp*	−0.386 ***	−0.112 **	−0.097 ***	−0.402 ***
	(−5.31)	(−2.53)	(−3.56)	(−3.84)
*edu*	0.020	0.060 *	0.104 ***	−0.082
	(0.40)	(1.72)	(4.61)	(−1.13)
*gov*	1.229 ***	0.623 ***	0.714 ***	1.368 ***
	(4.31)	(3.06)	(4.67)	(3.60)
*fina*	−0.011	−0.009	0.025	0.099 **
	(−0.42)	(−0.31)	(1.49)	(2.24)
*fdi*	0.036 ***	0.008	−0.002	0.039 ***
	(4.51)	(1.14)	(−0.43)	(3.51)
*trade*	0.055 ***	0.022	0.011	0.100 ***
	(2.71)	(1.61)	(1.13)	(3.39)
*Year FE*	Yes	Yes	Yes	Yes
*City FE*	Yes	Yes	Yes	Yes
*Constant*	3.120 ***	1.227 ***	1.010 ***	3.287 ***
	(4.21)	(2.77)	(3.49)	(3.39)
*N*	1699	2165	2735	1227
*R^2^*	0.203	0.218	0.244	0.198

*Notes: t*-statistics in parentheses, *** *p* < 0.01, ** *p* < 0.05, * *p* < 0.1.

**Table 7:** Mediation effect test.

	(1)	(2)	(3)	(4)
	Number of Innovations		Innovation Quality	
	*uco2*	*uco2*	*uco2*	*uco2*
*kdazh*	−0.030 **	−0.025 **	−1.210 ***	−1.250 ***
	(−2.41)	(−2.02)	(−4.97)	(−5.16)
*kdzg*	0.144	0.102	0.053	0.056
	(1.39)	(1.00)	(1.36)	(1.45)
*indushige*	0.089 ***	0.102 ***	−0.089	−0.061
	(6.00)	(6.65)	(−0.76)	(−0.52)
*lngdp*		−0.255 ***		−0.216 ***
		(−6.95)		(−5.99)
*edu*		0.021		0.031
		(0.77)		(1.13)
*gov*		0.788 ***		0.836 ***
		(4.67)		(4.95)
*fina*		0.041 **		0.053 ***
		(2.08)		(2.72)
*fdi*		0.016 ***		0.017 ***
		(2.91)		(3.10)
*trade*		0.031 ***		0.039 ***
		(2.69)		(3.45)
*Year FE*	Yes	Yes	Yes	Yes
*City FE*	Yes	Yes	Yes	Yes
*Constant*	0.311 ***	1.957 ***	0.772 ***	1.945 ***
	(3.99)	(5.46)	(32.19)	(5.38)
*N*	3962	3962	3962	3962
*R^2^*	0.162	0.189	0.159	0.185

*Notes: t*-statistics in parentheses, *** *p* < 0.01, ** *p* < 0.05.

**Table 8:** Policy linkage effects.

	(1)	(2)
	*uco2*	*uco2*
*didld*	−0.135 ***	−0.129 ***
	(−3.99)	(−3.77)
*lngdp*		−0.185 ***
		(−5.18)
*edu*		0.040
		(1.45)
*gov*		0.972 ***
		(5.81)
*fina*		0.052 ***
		(2.63)
*fdi*		0.017 ***
		(3.13)
*trade*		0.038 ***
		(3.32)
*Year FE*	Yes	Yes
*City FE*	Yes	Yes
*Constant*	0.762 ***	1.621 ***
	(37.75)	(4.53)
*Observations*	3962	3962
*R-squared*	0.151	0.176

*Notes: t*-statistics in parentheses, *** *p* < 0.01.

## Data Availability

The datasets used and analyzed in the current study are available from the corresponding author upon reasonable request.
